# Correlational Analysis of neck/shoulder Pain and Low Back Pain with the Use of Digital Products, Physical Activity and Psychological Status among Adolescents in Shanghai

**DOI:** 10.1371/journal.pone.0078109

**Published:** 2013-10-11

**Authors:** Zhi Shan, Guoying Deng, Jipeng Li, Yangyang Li, Yongxing Zhang, Qinghua Zhao

**Affiliations:** Department of Orthopaedic Surgery, Shanghai First People's Hospital, School of Medicine of Shanghai Jiao Tong University, Shanghai, China; University of South Australia, Australia

## Abstract

**Purpose:**

This study investigates the neck/shoulder pain (NSP) and low back pain (LBP) among current high school students in Shanghai and explores the relationship between these pains and their possible influences, including digital products, physical activity, and psychological status.

**Methods:**

An anonymous self-assessment was administered to 3,600 students across 30 high schools in Shanghai. This questionnaire examined the prevalence of NSP and LBP and the level of physical activity as well as the use of mobile phones, personal computers (PC) and tablet computers (Tablet). The CES-D (Center for Epidemiological Studies Depression) scale was also included in the survey. The survey data were analyzed using the chi-square test, univariate logistic analyses and a multivariate logistic regression model.

**Results:**

Three thousand sixteen valid questionnaires were received including 1,460 (48.41%) from male respondents and 1,556 (51.59%) from female respondents. The high school students in this study showed NSP and LBP rates of 40.8% and 33.1%, respectively, and the prevalence of both influenced by the student’s grade, use of digital products, and mental status; these factors affected the rates of NSP and LBP to varying degrees. The multivariate logistic regression analysis revealed that Gender, grade, soreness after exercise, PC using habits, tablet use, sitting time after school and academic stress entered the final model of NSP, while the final model of LBP consisted of gender, grade, soreness after exercise, PC using habits, mobile phone use, sitting time after school, academic stress and CES-D score.

**Conclusions:**

High school students in Shanghai showed high prevalence of NSP and LBP that were closely related to multiple factors. Appropriate interventions should be implemented to reduce the occurrences of NSP and LBP.

## Introduction

Neck/shoulder pain (NSP) and low back pain (LBP) have recently been identified as problems in many countries. As residents of the largest developing country, people in China also suffer from NSP and LBP. The occurrence of NSP and LBP places a heavy burden on both the individual and society in general. In addition, many studies have shown that the prevalence of NSP and LBP is high among adolescents [1–5]. A survey in Finland showed that NSP occurred at least once a week in approximately 26% of 14- to 18-year-olds; LBP also occurred in 12% of this population [1]. According to data published by the Ministry of Education of the People’s Republic of China, there were 2,358 institutions of higher education in 2010, and the population participating in the college entrance examination was 9.46 million. Given the combined effects of the large population base of China, its difficult employment environment and other factors, high school students in China face difficult challenges. Although levels of education have increased, appropriate methods of guidance and psychological counseling are still relatively insufficient. Thus, students tend to sacrifice their sleep and activity time as well as extend their study time to meet certain requirements. For adolescents, these factors may lead to depression, a lack of exercise, and skeletal-muscle dysfunction. Additionally, youths are usually highly interested in new technology; this population has high rates of personal computer (PC), mobile phone, tablet computer (tablet), and other electronic product usage.

In recent years, numerous studies have shown that the prevalence of NSP and LBP in adolescents is increasing [6,7]; these prevalence rates are especially high in girls [8,9]. Furthermore, the occurrence of NSP and LBP is related to many factors, including depression, physical activity, and lifestyle [3,8–11]. Although China is an important region in Asia, there is a lack of data concerning the prevalence of NSP and LBP in Chinese adolescents and their influencing factors. Moreover, there is limited research concerning mobile phone use and musculoskeletal diseases, and no research has concentrated on tablet usage. Therefore, our survey is significant and specifically designed to assess this pain in Chinese youth.

This study investigated the relationship between the prevalence of NSP and LBP in adolescents and physical activity, psychological pressure incommensurate with grade level, and the use of digital products.

## Methods

### Ethics Statement

The Ethics Committee at the School of Medicine, Shanghai Jiaotong University reviewed and approved this project. Written informed consent was obtained from the students' guardians.

### Participants

An epidemiological, anonymous self-assessment was administered to high school students in Shanghai, China. Thirty college students majoring in clinical medicine at the School of Medicine, Shanghai Jiaotong University, who had previously received epidemiological survey training, distributed and collected these questionnaires during the second semester of the 2011-2012 school year. Thirty schools were randomly selected from among the 237 high schools registered in Shanghai, China to participate in the study. In each school, 120 questionnaires were distributed to first-year, second-year and third-year high school students (aged 15-17 years, 16-18 years, and 17-19 years, respectively). These students were randomly selected using their student ID numbers. The paper questionnaires were distributed, completed and collected on each site within 20 minutes. While the participants filled out the survey, they were able to ask the administrators for assistance if they had any problems with the questionnaire. 

### Questionnaire

The questionnaire mainly examined the prevalence of NSP and LBP, physical activity after school, study pressure and the use of PCs, mobile phones, and tablets. The CES-D (Center for Epidemiological Studies Depression) scale was also included in the survey. Prior to administering this large-scale survey, the applicability of the questionnaire and the students’ comprehension of it were assessed using a small-scale test among freshmen in the School of Medicine, Shanghai Jiaotong University. After our pre-test, factor analysis was used to optimize the questionnaire, with the result of the factor analysis a KMO of 0.559, therefore the construct validity is considered sufficient. The reliability of the questionnaire was evaluated using a test-retest study which repeated measures on 150 subjects two weeks later. The questionnaire was found to be reliable with a coefficient of between 0.887 and 0.980.The questionnaire components were as follows:

#### Demographic information

This section assessed gender (male or female) and high school year (1, 2, or 3).

#### NSP and LBP

The regions of NSP and LBP were explicitly described through a diagram in the questionnaire. Specifically, NSP and LBP were assessed using the following question: "For the past six months, I have felt discomfort in my neck/shoulder or lower back (excluding menstrual discomfort for girls)." The answer options were: "almost never" (less than once a month), "occasionally" (1-3 times a month), "often" (1-3 times a week), and "always" (more than three times a week). "Often" and "always" were considered to denote the presence of NSP and LBP clinical symptoms. Additionally, preexisting musculoskeletal conditions were inquired about in this section.

#### Digital product

The question concerning PC usage was "How long do you use a PC to study or for entertainment each day (< 0.5 h, 0.5-1 h, 1-1.5 h, or > 1.5 h)?" In addition, data regarding the type of the PC in use (desktop or laptop), its relative height, the eye-to-computer screen distance, and whether the respondent sat in front of the computer with little movement for a significant duration (i.e., 1.5 h) were collected. The first question regarding mobile phone and tablet use was "Do you use a mobile phone/ tablet?" Those who answered "yes," were asked additional questions concerning posture, average daily use time, and eye-to-screen distance while using a mobile phone/tablet. 

#### Extracurricular activities

The assessment of extracurricular activities primarily included the intensity of regular physical activity, the frequency of physical activity each week, the average time of each physical activity, and whether muscle soreness occurred after exercise. In addition, the two statements, "I consciously physically exercise" and "I spend sufficient time on physical activities and feel satisfied about it" were used to assess the students’ feelings toward physical activity.

#### Academic pressure and mental status

This section included the statements, "I am satisfied with my studies every day," "I am relatively satisfied with my academic performance," and "I feel depressed while studying" to assess the academic pressure felt by the responding students. At the end of the questionnaire, a CES-D was used to assess depression. The CES-D includes 20 self-assessment questions to measure depression levels among respondents [12]. This test has been proved to be applicable to adolescents [13]. The CES-D was not compulsory, and it has a maximum possible score of 60 points with a score greater than 16 points denoting depressive symptoms in the adolescent.

### Data analysis

A chi-square test was used to compare the prevalence of NSP and LBP. The correlations among after-school physical activities, academic stress, and psychological status as well as the use of PCs, mobile phones, and tablets were analyzed using univariate analyses and multivariate logistic regression models with a forward stepwise selection method. The possible covariates were each examined in the multivariate logistic regression; the removal limit for variables was 0.10. Gender and grade were considered as potential confounders and were forced into the model to reduce confounding. The results are presented below as odds ratios (OR) and 95% confidence intervals (95% CIs) below. This study defined differences associated with a P < 0.05 as significant. All data analyses were conducted using SPSS 19.0.

## Results

Of the 3,600 questionnaires, 3,397 (94.4%) were collected, and 3,016 (83.8%) were confirmed to be valid. The remaining questionnaires were discarded due to their incomplete answers. The following data analyses were based on the valid questionnaires.

The overall prevalence of NSP and LBP among the high school students in this study were 40.8% and 33.1%, respectively. Significantly more girls (693 of 1,556; 44.5%) reported having NSP compared with boys (537 of 1,460; 36.8%). In addition, significantly more girls (563 of 1,556; 36.2%) reported having LBP compared to boys (435 of 1,460; 29.8%). The prevalence of NSP and LBP across high school years tended to increase with the grade level (p < 0.05; Table 1). Forty-two respondents reported having preexisting musculoskeletal diseases, with 41 of them being fractures of limbs or ribs, only one respondent reported neck/shoulder pain, which was associated with a recent ulna fracture (and was potentially caused by a sling). Additionally, one respondent reported having ankylosing spondylitis with low back pain, but was not quite sure about the actual situation.

**Table 1:** Sociodemographic Factors, Physical Activity and the Risks of NSP and LBP in High School Students.

	N	% With NSP	P Value	NSP OR (95% CI)	% With LBP	P Value	LBP OR (95% CI)
Gender			0			0	
Male	1460	36.8%		0.72 (0.62-0.84)	29.8%		0.75 (0.64-0.87)
Female	1556	44.5%		1	36.2%		1
High school year			0.05			0.08	
1	1267	39.0%		1	31.6%		1
2	1334	40.9%		1.08 (0.92-1.27)	33.1%		1.07 (0.91-1.26)
3	415	45.8%		1.32 (1.06-1.65)	37.6%		1.30 (1.03-1.64)
Intensity of regular physical activities			0.24			0.01	
Light	835	42.6%		1	37.1%		1
Moderate	998	41.3%		0.95 (0.79-1.14)	32.8%		0.83 (0.68-1.00)
Heavy	1183	39.0%		0.86 (0.72-1.03)	30.5%		0.74 (0.62-0.90)
Frequency of weekly exercise			0.06			0.02	
Once a week or less	893	43.8%		1	36.8%		1
1-4 times a week	1518	38.9%		0.82 (0.69-0.97)	30.7%		0.76 (0.64-0.90)
5-7 times a week	512	39.8%		0.85 (0.68-1.06)	33.6%		0.87 (0.69-1.09)
More than 5-7 times a week	93	47.3%		1.15 (0.75-1.77)	33.3%		0.86 (0.55-1.35)
Average time of each exercise			0.04			0.04	
< 0.5 h	1152	42.8%		1	35.6%		1
0.5 h-1 h	1544	39.2%		0.86 (0.74-1.01)	30.7%		0.80 (0.68-0.94)
1 h-2 h	229	37.1%		0.79 (0.59-1.06)	34.5%		0.95 (0.71-1.28)
> 2 h	91	50.5%		1.37 (0.89-2.10)	38.5%		1.13 (0.73-1.76)
I consciously physically exercise			0.51			0.01	
No	1514	41.3%		1	35.2%		1
Yes	1502	40.1%		0.95 (0.82-1.10)	31.0%		0.83 (0.71-0.96)
I spend sufficient time on physical activities, and I feel satisfaction about it			0			0.01	
No	1651	43.3%		1	35.0%		1
Yes	1365	37.7%		0.79 (0.68-0.92)	30.8%		0.83 (0.71-0.96)
How often do you feel soreness after exercise?			0			0	
Almost never	901	31.7%		1	26.7%		1
Occasionally	1565	41.2%		1.50 (1.27-1.79)	31.2%		1.24 (1.03-1.49)
Often	438	54.1%		2.54 (2.01-3.21)	47.5%		2.48 (1.95-3.14)
Always	112	55.4%		2.67 (1.79-3.97)	54.5%		3.28 (2.20-4.89)

NSP = neck/shoulder pain; LBP = lower back pain; OR = ORs after univariate logistic regression.

51.9% of students reported using PCs for more than half an hour per day. Laptop PC users showed a higher prevalence of NSP and LBP compared to desktop PC users. Height and eye-to-screen distance was not related to the degree of LBP, but the latter variable was related to the occurrence of NSP (Table 2). Among all respondents, 85.4% were mobile phone users who were less likely to suffer from LBP, but a period of mobile phone use longer than 2 hours per day were related to a significant increase in the prevalence of NSP and LBP (p < 0.05, Table 3). The use of a tablet (1,067 of 3,016, 35.4%) significantly increased the incidence of NSP. This result was primarily due to the posture and eye-to-screen distance maintained while using a tablet. The use of a tablet was not related to the incidence of LBP; however, long-term use and small eye-to-screen distances were significantly related to increases in the incidence of LBP (Table 4).

**Table 2:** PC use and the Risks of NSP and LBP in High School Students.

	N	% With NSP	P Value	NSP OR (95% CI)	% With LBP	P Value	LBP OR (95% CI)
Average daily PC use time			0.21			0.03	
< 0.5 h	1452	40.6%		1	32.3%		1
0.5 h-1 h	660	37.9%		0.89 (0.74-1.08)	29.8%		0.89 (0.73-1.09)
1 h-1.5 h	528	42.2%		1.07 (0.87-1.31)	36.2%		1.19 (0.96-1.46)
> 1.5 h	376	44.1%		1.16 (0.92-1.45)	37.5%		1.26 (0.99-1.59)
I sat in front of a PC without moving much for more than 1.5 h			0.01			0.08	
No	1812	38.9%		1	31.8%		1.15 (0.99-1.34)
Yes	1204	43.6%		1.22 (1.05-1.41)	35.0%		1.15 (0.99-1.34)
Type of PC used			0			0	
Laptop	1145	45.3%		1	36.6%		1
Desktop	1871	38.5%		0.76 (0.65-0.88)	31.2%		0.79 (0.67-0.92)
Height of the PC screen			0.41			0.1	
Eyes above the midpoint of screen	799	41.4%		1	34.5%		1
Eyes approximate the midpoint of screen	2006	40.1%		0.95 (0.80-1.12)	32.0%		0.90 (0.75-1.06)
Eyes below the midpoint of screen	211	44.5%		1.14 (0.84-1.54)	38.4%		1.18 (0.86-1.62)
Eye-to-screen distance while using a PC			0			0.07	
< 20 cm	280	36.4%		1	39.3%		1
20 cm-25 cm	832	36.7%		1.01 (0.76-1.34)	31.1%		0.70 (0.53-0.93)
25 cm-30 cm	1051	44.3%		1.39 (1.06-1.83)	32.3%		0.74 (0.56-0.97)
> 30 cm	853	41.7%		1.25 (0.95-1.65)	34.0%		0.80 (0.60-1.05)

NSP = neck/shoulder pain; LBP = lower back pain; OR = ORs after univariate logistic regression.

**Table 3:** Mobile phone use and the Risks of NSP and LBP in High School Students.

	N	% With NSP	P Value	NSP OR (95% CI)	% With LBP	P Value	LBP OR (95% CI)
Mobile phone use			0.79			0.01	
No	441	40.1%		1	38.5%		1
Yes	2575	40.9%		1.03 (084-1.27)	32.2%		0.76 (0.61-0.93)
Posture while using a mobile phone							
Standing	1001	42.0%	0.37	1.08 (0.92-1.27)	35.8%	0	1.31 (1.11-1.55)
Lying	755	46.9%	0	1.42 (1.20-1.68)	35.9%	0	1.27 (1.06-1.52)
Semi-reclining	732	42.1%	0.45	1.07 (0.90-1.28)	31.8%	0.85	0.98 (0.82-1.18)
Sitting	1224	35.9%	0	0.67 (0.58-0.79)	30.4%	0.07	0.86 (0.73-1.01)
Average daily mobile phone use time			0			0	
< 1 h	1069	38.4%		1	27.0%		1
1 h-1.5 h	724	42.1%		1.17 (0.97-1.42)	34.8%		1.44 (1.18-1.77)
1.5 h-2 h	321	35.8%		0.90 (0.69-1.16)	31.2%		1.22 (0.93-1.60)
> 2 h	461	48.2%		1.493 (1.20-1.86)	40.6%		1.84 (1.46-2.32)
Eye-to-screen distance while using mobile phone			0.02			0	
< 10 cm	432	46.5%		1	42.4%		1
10 cm-15 cm	1053	38.7%		0.72 (0.58-0.91)	28.8%		0.55 (0.44-0.69)
15 cm-20 cm	541	42.7%		0.86 (0.66-1.11)	28.7%		0.55 (0.42-0.71)
> 20 cm	549	38.8%		0.73 (0.56-0.94)	34.1%		0.70 (0.54-0.91)

NSP = neck/shoulder pain; LBP = lower back pain; OR = ORs after univariate logistic regression.

**Table 4:** Tablet use and the Risks of NSP and LBP in High School Students.

	N	% With NSP	P Value	NSP OR (95% CI)	% With LBP	P Value	LBP OR (95% CI)
I use a tablet			0.01			0.87	
No	1949	38.9%		1	33.2%		1
Yes	1067	44.1%		1.24 (1.07-1.45)	32.9%		0.99 (0.84-1.16)
Posture while using a tablet							
Standing	135	45.9%	0.71	1.09 (0.76-1.56)	31.1%	0.7	0.91 (0.62-1.34)
Lying	232	46.6%	0.41	1.13 (0.85-1.52)	39.7%	0.01	1.46 (1.08-1.98)
Semi-reclining	315	44.4%	0.95	1.02 (0.78-1.33)	31.7%	0.62	0.93 (0.70-1.23)
Sitting	545	41.5%	0.07	0.80 (0.63-1.02)	29.4%	0.01	0.72 (0.56-0.93)
Average daily tablet use time			0.21			0.18	
< 1 h	37	43.2%		1	24.3%		1
1 h-1.5 h	347	45.8%		0.56 (0.56-2.20)	36.0%		1.75 (0.80-3.83)
1.5 h-2 h	567	41.6%		0.48 (0.48-1.83)	30.7%		1.38 (0.64-2.98)
> 2 h	116	51.7%		0.67 (0.67-2.96)	37.1%		1.83 (0.79-4.25)
Eye-to-screen distance while using tablet			0			0	
< 15 cm	164	57.3%		1	48.8%		1
15 cm-20 cm	358	39.1%		0.48 (0.33-0.70)	29.6%		0.44 (0.30-0.65)
20 cm-25 cm	301	40.5%		0.51 (0.35-0.75)	29.2%		0.43 (0.29-0.64)
> 25 cm	244	47.1%		0.66 (0.45-0.99)	31.6%		0.48 (0.32-0.73)

NSP = neck/shoulder pain; LBP = lower back pain; OR = ORs after univariate logistic regression.

Students who consciously engaged in physical activities had a lower prevalence of NSP and LBP compared to those who lacked physical exercise; the difference in the incidence of LBP was significant (P < 0.05). In addition, those who reported more satisfaction with physical exercise had a significantly lower prevalence of NSP and LBP (P < 0.05). 39.2% of the students who regularly participated in heavy exercise showed a lower prevalence of LBP and NSP than those involved in moderate or light exercise, but this difference was not significant (P > 0.05). The frequency of exercise was also associated with the prevalence of NSP and LBP; the lowest prevalence of these conditions were found among students who engaged in physical activity 1-4 times weekly. The group that exercised for approximately an hour each day showed significantly less NSP and LBP than those who did so for longer or shorter periods (Table 1). Additionally, 26.1% of students reported that their sitting time out of school was more than three hours every day; these students were significantly more likely to have NSP (Table 5). 

**Table 5:** Depressive Symptoms, Studying Stress and the Risks of NSP and LBP in High School Students.

	N	% With NSP	P Value	NSP OR (95% CI)	% With LBP	P Value	LBP OR (95% CI)
Continue sitting after school every day			0			0	
< 3 h	2230	36.2%		1	30.9%		1
> 3 h	786	53.7%		2.04 (1.73-2.41)	39.2%		1.44 (1.21-1.70)
I feel depressed			0			0	
No	2059	35.6%		1	26.7%		1
Yes	957	51.7%		1.93 (1.66-2.26)	46.9%		2.43 (2.07-2.85)
I’m satisfied with studying every day			0.03			0.01	
No	1746	42.4%		1	34.9%		1
Yes	1270	38.5%		0.85 (0.73-0.99)	30.6%		0.82 (0.70-0.96)
I am satisfied with my academic performance			0			0	
No	1540	45.1%		1	37.5%		1
Yes	1476	36.2%		0.69 (0.60-0.80)	28.5%		0.66 (0.59-0.77)
CES-D score			0			0	
< 16	1994	38.8%		1	29.1%		1
≥ 16	767	46.0%		1.34 (1.14-1.59)	43.9%		1.91 (1.61-2.27)

NSP = neck/shoulder pain; LBP = lower back pain; OR = ORs after univariate logistic regression.

Students with higher levels of satisfaction in their daily studies and academic performance showed lower rates of NSP and LBP; those who felt depressed due to academic pressure showed a higher prevalence of NSP and LBP (P < 0.05). In addition, 2,761 of 3,016 students finished the CES-D, and 27.8% of those students showed depression scores greater than 16; scores greater than 16 were significantly related to the occurrences of NSP and LBP (Table 5).

The multivariate models of NSP and LBP are presented in Table 6 and Table 7. Gender, grade, soreness after exercise, PC using habits, tablet use, sitting time after school and academic stress entered the final model of NSP, while the final model of LBP consisted of gender, grade, soreness after exercise, PC using habits, mobile phone use, sitting time after school, academic stress and CES-D score. 

**Table 6:** Multivariate model for association between NSP in High School Students.

	NSP OR (95% CI)
Gender: Female	1.293(1.108-1.509)
High school year	
1	0.931(0.789-1.098)
2	1
3	1.335(1.056-1.687)
Frequency of feeling soreness after exercise	
Almost never	1
Occasionally	1.464(1.223-1.752)
Often	2.211(1.730-2.825)
Always	2.069(1.365-3.137)
Often sitting in front of a PC without moving much for more than 1.5 hours	1.243(1.063-1.454)
Type of PC used: laptop	1.366(1.167-1.599)
Eye-to-screen distance while using a PC	
< 20 cm	1
20 cm-25 cm	1.068(0.794-1.436)
25 cm-30 cm	1.471(1.103-1.962)
> 30 cm	1.437(1.069-1.931)
Tablet use	1.311(1.117-1.538)
Continue sitting after school every day over 3 hours	1.854(1.561-2.202)
Feeling depressed	1.821(1.546-2.146)
Not satisfied with academic performance	1.390(1.191-1.622)

NSP = neck/shoulder pain; OR = ORs after multiple logistic regression with forward stepwise selection.

**Table 7:** Multivariate model for association between LBP in High School Students.

	LBP OR (95% CI)
Gender: Female	1.296(1.102-1.523)
High school year	
1	0.965(0.811-1.147)
2	1
3	1.309(1.029-1.666)
Frequency of feeling soreness after exercise	
Almost never	1
Occasionally	1.321(1.093-1.598)
Often	2.200(1.714-2.823)
Always	2.575(1.697-3.909)
Type of PC used: laptop	1.339(1.136-1.578)
Mobile phone use	1.311(1.048-1.639)
Sitting time (except in school)every day over 3 hours	1.246(1.042-1.490)
Feeling depressed	2.039(1.724-2.412)
Not satisfied with academic performance	1.305(1.110-1.534)
CES-D score ≥ 16	1.681(1.420-1.991)

LBP = lower back pain; OR = ORs after multiple logistic regression with forward stepwise selection.

## Discussion

The results of this survey are consistent with numerous previous and similar studies suggesting that the prevalence of musculoskeletal diseases such as LBP and NSP is considerable among high school students. The occurrence of NSP and LBP in Shanghai adolescents is related to several factors including gender, grade, soreness after exercise, digital device use, sitting time after school and personal emotions.; but is not related to physical exercise.

Of the 3,600 questionnaires, 3,016 were analyzed. The overall prevalence of NSP and LBP was 40.8% and 33.1%, respectively. These results are significantly higher than those in a study on adolescent musculoskeletal system diseases in the Netherlands [3]; this divergence might be related to the heavy academic pressures placed on high school students in China. Our survey data also revealed that the prevalence of NSP and LBP increased with the high school students' grade; the multivariate logistic regression analysis revealed that the third year of high school is a major risk factor for both NSP and LBP. This result might be closely related to the academic and psychological pressure placed on final-year high school students due to the approaching college entrance exams in China; it may also relate to the increase in sedentary states and the reduction of physical activity caused by the academic burden. Our results showed that the prevalence of NSP and LBP were significantly higher in girls compared to boys, which is consistent with the findings of numerous domestic and foreign surveys on adults and adolescents [3,9,10]. We speculate that this finding may be related to the following reasons: (a) boys always have a higher pain threshold than girls [14,15]; (b) the special hormonal changes in girls during puberty [16]; (c) the lower physical activity levels of girls compared to boys; (physical activity is reported to be a positive factor in preventing musculoskeletal diseases; however, the current research did not find an association between physical activity and NSP or LBP) [17]; (d) the tendency of girls to have more mental stress than boys, where stress has been found to correlate with musculoskeletal diseases [3,18,19]; and (e) the heritability of NSP in girls is higher than in boys [20]. 

Various surveys of adults and adolescents have shown that PC use can increase the incidence of LBP [1,21–24]. In a small-scale study, Jacobs and Baker [25] found that PC-use time and the occurrence of musculoskeletal pain were correlated. Hakala et al. [1] also showed that PC use increases the prevalence of NSP and LBP in adolescents. In contrast, the results of this study did not find a correlation between PC use time and NSP or LBP, which is consistent with the findings of Harreby et al. [26] and Diepenmaat et al. [3] who also did not find a significant correlation between PC use and LBP. The disagreement of different survey results might be due to inconsistent NSP and LBP definitions. Our research used more stringent definitions of NSP and LBP than those used by Jacobs and Baker. Moreover, as Faucett and Rempel [27] found, self-assessed PC-use time is often longer than actual usage, so the PC use time in some research studies might be inaccurate. In addition, our findings are from the specific urban situation of Shanghai, China, where there is no special necessity for high school students to use a PC for work, and students usually do not have much time to use a PC to access the Internet or play games due to the daily, heavy school workload. Interestingly, the desktop PC users showed significantly less NSP and LBP compared with laptop PC users. We believe that the more flexible placement of desktop PC devices (e.g., the screen or the keyboard can be set in more comfortable places) can provide a more natural and comfortable posture for desktop PC users, thereby reducing the occurrence of discomfort. 

Our study showed that many high school students in Shanghai use mobile phones, and the mobile phone users showed a significantly lower prevalence of LBP. Previous studies regarding the correlation between mobile phone use and NSP and LBP in adolescents is limited. Hakala et al. [1] suggested that mobile-phone use is not significantly correlated with the prevalence of NSP or LBP. We believe that it's easier for mobile phone users to establish a comfortable posture when making a call compared to wired-phone users, who sometimes have to remain in static postures while answering the phone. As a recent study showed, a static posture can increase bone and muscle stress around the waist and neck and is closely related to NSP and LBP [28]. 

Since January 2010, when the first generation iPad was released, tablets have become widely used around the world. Still, there is no research available examining the effect of tablet use on the musculoskeletal system. Our survey shows that the tablet, which is significantly correlated with a high prevalence of NSP, has a certain market penetration rate among high school students in Shanghai. The posture of most people who use a tablet is similar to their reading posture. Thus, the relationship between tablet use and NSP might be similar to that between an improper reading posture and NSP [29,30]. To some extent, tablet use increases the likelihood of triggering NSP compared to an improper reading posture because a tablet often needs one hand to operate the touch screen; thus, this use can lead to an uneven bilateral shoulder level, a bilateral force asymmetry in the body, and incorrect posture. An uneven shoulder level is one of the unhealthy postures that causes musculoskeletal pain [29]. Additionally, the entertainments on tablets might be more attractive than traditional books. In addition, smart phones such as iPhones, Android phones, and Windows phones have become important parts of the mobile phone market in China. Smart phone users, especially adolescents, may have different use habits compared to traditional mobile phone users; however, our questionnaire did not distinguish between these groups of phone users. The method of using a smart phone is somewhat similar to that of using a tablet, so the data related to tablet use might serve as a reference to the study of smart phone use and musculoskeletal pain.

Some studies have suggested that people who exercise regularly have a higher prevalence of NSP and LBP [2,31]. However, the multivariate model of this study only confirmed the relationship between sports injuries and NSP and LBP in which students with soreness after physical exercise were more susceptible to NSP and LBP. In addition, univariate analyses showed that approximately one hour of physical exercise is the amount most likely to prevent NSP and LBP. It is difficult to quantify the exercise effect when the exercise time is short; on the other hand, long exercise durations are more likely to be associated with sports-related injuries. The correlations between physical exercise injury and the intensity and duration of the exercise must still be clarified with future research. Based on our results, the correct amount, as well as the reasonable style and intensity of physical exercise, are all very important to the health of the musculoskeletal system in adolescence.

Our study showed that mental stress is related to a high prevalence of NSP and LBP. This result is consistent with the findings of numerous studies of youth [3,18,19]. In adulthood, studies have shown that a depressed mental state can predict LBP and NSP even without earlier symptoms [32]. According to our research, students with higher levels of satisfaction toward everyday learning showed less NSP and LBP. Moreover, feelings of depression entered the final multivariate model of the prevalence of both NSP and LBP, which is consistent with the findings of numerous other studies [1,2]. The college entrance exam in China plays an important role in the future plans of high school students; thus, Chinese high school students are generally under a considerable degree of psychological pressure. This circumstance might also explain our results that the prevalence of NSP and LBP in Chinese high school students was significantly higher than previously reported byforeign studies. (In our study, the prevalence of NSP and LBP were 40.8% and 33.1%, respectively, with 46.0% participants having a CES-D score greater than 16, while the corresponding data in a study from the Netherlands were 11.5%, 7.5%, and 22.3%, respectively). Unsurprisingly, the students who sat as they studied for more than 3 hours each day after school were significantly more likely to have NSP. Other research has also shown that maintaining a sedentary position, especially with an improper sitting posture, can trigger NSP [29]. The CES-D questionnaire data showed that students with LBP had a significantly higher depression score than those without LBP, but NSP did not present this difference. The multivariate logistic regression analysis also revealed that a CES-D score greater than 16 was a risk factor for LBP. The difference might be due to the higher prevalence of NSP, with more students experiencing NSP, they may be more willing to reduce the pain, especially those students who have depressive feelings. Additionally, during our survey, we found that the students were more willing to ask questions about NSP than LBP. Several studies also revealed the positive correlation between CES-D scores and the prevalence of musculoskeletal diseases [33,34], Psychological treatments can have positive effects on the musculoskeletal systems pain in adolescents, which suggests that depressive symptoms and stress are more likely to be causes rather than consequences of musculoskeletal pain. A randomized, controlled trial of Dutch adults indicated that cognitive behavioral therapy (a therapy widely accepted in clinics to treat depression) can relieve a variety of pains that are difficult to explain, including musculoskeletal pain [35,36]. During the 6- to 12-month follow-up period, the treatment group had a higher cure rate and less symptoms compared to the control group. Eccleston et al. [37] have confirmed the therapeutic effect of cognitive behavioral therapy with regard to treating chronic pain in adolescents.

The large sample size and randomly picked sample are among the primary strengths of this study. However, the current research is limited by its cross-sectional design, which is less sufficient in revealing the causality of the factors. Our results may have been affected by survey bias because respondents with clinical manifestations of NSP and LBP were more likely to complete the questionnaire; However, an analysis of incomplete questionnaires showed that there were no systematic or statistically significant differences in the potential factors between incomplete and complete questionnaires. Physical activity was measured using validated questions but relied on self-report rather than on objective measurements, which may limit the findings. It was suggested that self-report might include large measurement errors for physical activities in minority groups [38]. However, this study measured the strength, frequency and length of physical activity, and had detailed options for each question, which provided good information on physical activity patterns. The test-retest results of the questions were also satisfactory; we therefore believe that this limitation is of minor importance. A prospective cohort study will be necessary in the future.

## Conclusions

High school students in China, especially girls and students in their final year, have significantly increased prevalence of NSP and LBP (≥ 4 days/month) compared with those reported in similar studies from other countries. The prevalence of NSP and LBP is significantly associated with PC-use habits, the use of mobile phones and tablets, academic stress, and depression. These associations suggest that providing comprehensive physical education, monitoring posture and the usage time of mobile phones and tablets, relieving academic pressure through a variety of methods, and adjusting high school student emotions are the keys to reducing NSP and LBP in high school students. The effect of the corresponding measures must still be determined with additional studies.
